# The use of statistical tools in field testing of putative effects of genetically modified plants on nontarget organisms

**DOI:** 10.1002/ece3.640

**Published:** 2013-06-19

**Authors:** Alexander V Semenov, Jan Dirk Elsas, Debora C M Glandorf, Menno Schilthuizen, Willem F Boer

**Affiliations:** 1 Department of Microbial EcologyCentre for Life Sciences, University of GroningenP.O. BOX 11103, 9700 CC, Groningen, The Netherlands; 2 National Institute of Public Health and the Environment, SEC/GMO OfficeP.O. Box 1, 3720 BA, Bilthoven, The Netherlands; 3 Naturalis Biodiversity CenterP.O. Box 9517, 2300 RA, Leiden, The Netherlands; 4 Resource Ecology GroupDroevendaalsesteeg 3a, 6708 PB, Wageningen UR, The Netherlands

**Keywords:** Environmental risk assessment, experimental design, field trials, generalized linear models

## Abstract

To fulfill existing guidelines, applicants that aim to place their genetically modified (GM) insect-resistant crop plants on the market are required to provide data from field experiments that address the potential impacts of the GM plants on nontarget organisms (NTO's). Such data may be based on varied experimental designs. The recent EFSA guidance document for environmental risk assessment (2010) does not provide clear and structured suggestions that address the statistics of field trials on effects on NTO's. This review examines existing practices in GM plant field testing such as the way of randomization, replication, and pseudoreplication. Emphasis is placed on the importance of design features used for the field trials in which effects on NTO's are assessed. The importance of statistical power and the positive and negative aspects of various statistical models are discussed. Equivalence and difference testing are compared, and the importance of checking the distribution of experimental data is stressed to decide on the selection of the proper statistical model. While for continuous data (e.g., pH and temperature) classical statistical approaches – for example, analysis of variance (ANOVA) – are appropriate, for discontinuous data (counts) only generalized linear models (GLM) are shown to be efficient. There is no golden rule as to which statistical test is the most appropriate for any experimental situation. In particular, in experiments in which block designs are used and covariates play a role GLMs should be used. Generic advice is offered that will help in both the setting up of field testing and the interpretation and data analysis of the data obtained in this testing. The combination of decision trees and a checklist for field trials, which are provided, will help in the interpretation of the statistical analyses of field trials and to assess whether such analyses were correctly applied.

We offer generic advice to risk assessors and applicants that will help in both the setting up of field testing and the interpretation and data analysis of the data obtained in field testing.

## Introduction

In field experiments on plant effects on the soil habitat, for instance with genetically modified (GM) plants, five components need consideration. These are the hypothesis with respect to the effects, the experimental design, experimental execution, statistical analysis, and data interpretation (Hurlbert 18). Obviously, the hypothesis, experimental design, and execution are of primary importance, as, if it is not sound by any criterion, even a well-conducted experiment may fail to bring any novelty. Importantly, the function of the statistics applied is to show the clarity, conciseness, and objectivity with which the results are presented and interpreted. Thus, statistical design, analyses, and interpretations are critical aspects of experimentation. And, if any statistical or interpretative errors are made, the data need to be reanalyzed, which is an achievable task, given that the proper data set is available.

There are differences in the statistical approaches used by investigators that perform field trials with GM plants to study potential impacts on so-called nontarget organisms (NTO's). Generally, the aim of such experiments is to make comparisons between the impacts of GM plants compared to those of their near-isogenic counterparts. Unfortunately, such field experiments often follow a flawed experimental design, such as the use of insufficient numbers of replicates or improper blocking (for instance, a field separated in several unequal parts). In addition, the statistical analyses are sometimes incorrectly chosen.

In this review, we examine the statistical approaches used in current studies on the effects of GM plants on NTOs in the field, mainly focusing on arthropods and other invertebrates. Such studies are characterized by several features, such as a high variation in the abundance of NTOs (in contrast to an analysis of species diversity) with, often, nonnormal distributions (Druart et al. 10; Hoss et al. 17; Oliveira-Filho et al. 27; Yamamori 39). It is important to state that the experimental design (e.g., the field lay-out, sample size, sampling method, number of (sub) samples, and replicates, and the way in which the treatments are randomized over the experimental units) defines how the data should be analyzed. In other words, an appropriate choice of the experimental design, taking into account all sources of variation and establishing replicate numbers on the basis of these, is primordial. As we deal with field studies, we will first examine the design of experiments with respect to the statistical requirements posed by the scientific question.

## Experimental Design

Depending on the purpose of the study, any field design for GM plant impact analysis should take into account the level of accuracy of the data needed in relation to the expected or observed variability. In particular, under- or overestimated impacts of, for example, GM plant cultivars should be avoided. For instance, a half-field (a field separated in two equal parts, a common example of blocking) design in comparison to paired fields has a high potential of reduction in environmental variability and so of measured impact. The reason is that two halves of a field are more likely to be similar in previous management, soil type, and surrounding habitat, than sites that are located away from each other (Perry et al. 28). However, care must be taken to avoid interferences between experimental units that are located so closely together. Establishment of separation distances, or buffer zones, according to agronomical rules (e.g., 50 m for rape and 6 m for beet; Perry et al. 28) between half-field units will help to minimize interference problems as well as to maintain the purity of crops.

### Structure of blocks, randomization, and replication

There are two fundamentally different experimental setups in which data can be obtained: (1) a designed experiment (control over experimental conditions and ability to vary these conditions) and (2) an observational study (in which conditions are beyond the control of the experimenter). For designed experiments, the main principles of randomization, replication, and across-unit homogeneity (blocking) are important. All designed experiments are usually set up as comparative experiments, in which a change in a variable is to be shown in relation to a cause (e.g., the presence of a transgene in the GM plant). A properly designed experiment must follow three rules/principles:

1. are randomly allocated to experimental units to neutralize the effects of location (or other uncontrolled factors, e.g., weather effects);
2. are sufficiently replicated to allow an adequate estimation of experimental error variance;
3. experimental units are (e.g., often, different fields or plots) grouped into homogeneous blocks prior to application of the treatment in order to minimize the impact of other controllable factors, such as differences in soil composition (Schabenberger and Pierce 43).

If a variable by which the experimental units should be blocked is not taken into account, the experimental design can lead to relatively large errors and thereby make it more difficult to find treatment effects. The resulting design might thus be inefficient as a result of this large error. Moreover, statistical tests that are applied might be lacking power. If treatments are replicated but not randomly assigned to experimental units, the data should be treated as observational, because the effect of location is not neutralized by randomization. Thus, some previously conducted NTO studies should be characterized as observational studies (e.g., Yamamori 39) due to the lack of (controlled) replicates and/or randomization.

**Figure 1:**
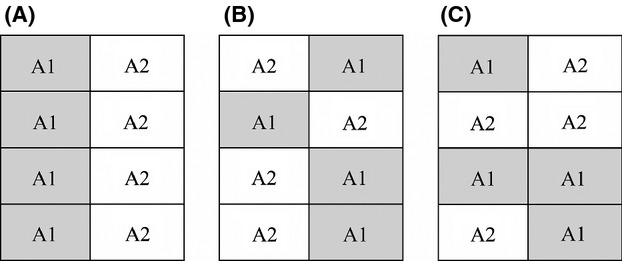
Linked to the type of randomization, treatment effects may interfere with nontreatment effects. For instance, in a completely randomized experimental block design, all replicates of treatment A1 may lie in the west of the field, whereas those of A2 lie in the East (A). In this case, wind or water flows from a certain direction might cause treatment differences by nontreatment effects. Such randomization might be done with a more balanced arrangement (B). East–west effects can be controlled by blocking (C) with a restriction for each treatment to appear two times in the east and two times in the west.

**Figure 2:**
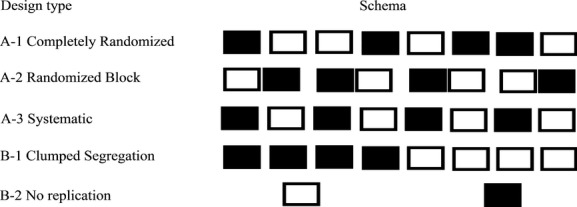
Several acceptable (A modes) and unacceptable ways of treatments (B modes) in a two-treatment experiment (shaded and unshaded). Each unit is assumed to have been treated independently of the other units in the same treatment (Hurlbert 18).

While blocking (Fig. 1) eliminates the effects of systematic factors in a well-designed experiment, randomization (Fig. 2) can neutralize the effects of unknown factors and allow to estimate treatment differences and variance components without systematic bias. On the other hand, replication does not necessarily lead to unbiased calculations of treatment effects (Schabenberger and Pierce 43).

### Experimental unit size

To determine the proper experimental design, measurements of variables (e.g., the density and dispersal rates of NTOs) should be taken at different locations in the experimental unit (e.g., a field) before an experiment is performed. A pilot study can be used for this. It is also possible to use general knowledge on the level of variation and possible distributions, for example, from other trials or from the literature (Table 1). These measurements will provide information about the appropriate buffer zones between plots and the level of expected variation in the field, thus indicating an optimal experimental design as well as the statistical approach to be used. Furthermore, it is important to know how many samples have to be taken for each field assessment, the minimally required sample size, and to determine the size of each sample (e.g., the size of the area to be sampled in one sampling). One has to balance the efforts in terms of allowing a higher number of samples per experimental unit or a lower one in exchange for more experimental units. However, a general rule of experimentation is that it is more efficient to have more experimental units with fewer samples per unit than fewer units with more samples. Clark et al. (9) compared the influence of herbicide management of a trial with herbicide-tolerant (GM) and conventional crops on local weed densities and indicated the effective number of samples which allowed distinguishing the effects of GM from those of non-GM crops. High variability in the values of variable indicators is usually counterbalanced by increasing numbers of samples, meaning that for indicators with lower variability, smaller numbers of samples can be used (Clark et al. 9). This is dependent on the size of the effect. Thus, a trade-off exists between an increase in the size of a sample (e.g., field size) and an increase in the sample number (e.g., replicates).

**Table 1:** Experimental unit (plot) size should be chosen in such a way that interference between plots is likely to be absent

Dispersal rate	Taxa	Appropriate size of the plots	References
Low	Snails; mites; flightless aphids; springtails; larval stages winged insects	25 m^2^	Schilthuizen and Lombaerts 45, 44; Gil et al. 15; Auclerc et al. 3; Lehmitz et al. 21.
Moderate	Adult spiders; adult soil-dwelling beetles (e.g., ground beetles); thrips	250 m^2^	den Boer 5; Liebherr 22; Bonte et al. 7; Morsello et al. 26.
Fairly high	Adult bugs; other (winged) beetles, adults; winged aphids	2500 m^2^	Smith King 34; Hazell et al. 16.
High	Bees; adult butterflies; adult flies; adult moths; juvenile spiders	25,000 m^2^	Feder et al. 13; Cameron et al. 8; Bonte et al. 7; Løjtnant et al. 23; Slatkin 46.

Distances (buffers) between fields should be at least the same as plot widths.

Such interference is likely to result partly from the fact that the individual dispersal distances of NTOs may overlap with more than a single plots, and thus effects of treatment in one plot may show up in a different plot. To choose the appropriate plot size, therefore, some rules of thumb may be applied based on characteristic rates of commonly studied NTO's. Note that studies on immobile larval stages would require a smaller plots size than those on mobile adults.

### Sample number and size

Sample numbers are restricted by various pragmatic limitations. Thus, they need to be handled in reasonable time and with reasonable investment of labor and money. The expected variation in the variable to be assayed must first be determined by analysis of a small number of samples, for instance, in a pilot experiment. Thus, the mean of the preliminary pool might be required to be within, for example, 10% of the real population mean. This 10% value is considered to be accurate enough for most purposes (Perry et al. 29).


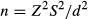


(where *n* – number of replicates; *Z* – probability [Z_0.05_ = 1.96]; *S*^2^ – error variance of samples, and *d*^2^ – margin of error for the plot) allows to calculate the required number of samples. This formula (1) is one of the most commonly used, although it might underestimate *n* (Kupper and Hafner 19). However, nowadays the number of replicates can be easily calculated by using any of many statistical packages that are able to estimate the required sample size under different experimental designs, taking into account the effect size, the variance, the degrees of freedom, and other factors (see also the Power analysis described below).

Sample numbers and sizes, in relation to the levels of variability, have thus to be adequate to test the assumption that there is no significant influence of GM plant cultivars as compared to non-GM ones. Many aspects of field experiments (e.g., experimental design and size of the unit of replication) have been discussed in the literature (Clark et al. 9; Perry et al. 28; Duan et al. 11), but there is no consensus as to how many replications are needed to detect a difference between a GM crop and its isogenic counterpart. This is obvious, as it depends on the magnitude of the putative difference, the plot size, the variability in the data, the design, the degrees of freedom, the trophic interactions, and other factors.

### Independency of samples and pseudoreplication

A fundamental assumption of all statistical analyses is that the data obtained from experimental studies represent independent observations of representative samplings from the population of interest (Andow 2). The measurements or observations are independent if the value of each observation is in no way influenced by, or related to, the value of other observations (LeBlanc 20). Hence, sampling a similar location twice, or even in different seasons or years can be a source of pseudoreplication.

**Figure 3:**
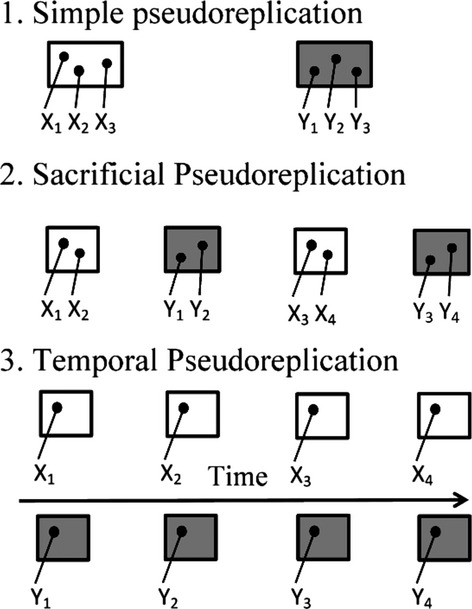
The figure (after Hurlbert 18) represents the three most common types of pseudoreplication. Shaded and unshaded boxes represent experimental units which receive different treatments. Each dot represents a sample or measurement. Pseudoreplication is a consequence (in each example) of statistical testing for a treatment effect by means of procedures which assume that the four data for each treatment have appeared from four independent experimental units. Important remark: example A cannot be analyzed properly, while B can, by taking the means for each unit.

Most models for statistical analysis require a particular level of true replication, which permits the estimation of variability within a treatment. Without estimating variability within treatments, it is impossible to perform statistical inference of differences. Repeated measures or pseudoreplicates are often confused with true replicates. Pseudoreplication represents a typical violation of the sample independency assumption. The term pseudoreplication (Hurlbert 18) refers to “the use of inferential statistics to test for treatment effects with data from experiments where either treatments are not replicated (though samples may be) or replicates are not statistically independent.” The following example illustrates the way this can occur (Fig. 3). It is sometimes possible to deal with pseudoreplication by using the mean of the subsamples or repeated measures in GLM analysis (discussed below). Doing statistical inference using pseudoreplicates rather than true replicates might cause an underestimation of variability. This will result in confidence intervals being too small and an inflated probability of a Type I error (falsely rejecting a true null hypothesis) occurs. For NTO field testing, it means that the chance to reject the null hypothesis is higher.

## Statistical Power

The power of a statistical test is related to the probability of distinguishing an effect (e.g., of a GM plant in comparison with its near-isogenic counterpart) as a function of the magnitude of the effect intended to be detected, the variability in the data and the number of values used to calculate the means (Andow 2). Therefore, all field studies should justify the sample sizes used (size of the sample and number of replicates). An analysis of statistical power (power analysis), as part of the analysis, is also a prerequisite of every study. A prospective statistical power analysis in order to confirm that the trial design fits the purpose of the study, is preferred above a retrospective power analysis (Andow 2; EFSA 12).

Power analyses also provide the confidence that the level of replication is neither too small to detect the effects that are present, nor too large to avoid that, unnecessarily, extra resources are used for trial experiments. It is important to apply difference tests (null hypothesis of no difference between the impact of a GM plant and a non-GM plant) for each experiment done to support an environmental risk assessment (ERA).

In practice, values of 70% (Prasifka et al. 42) and 80% (Perry et al. 28) are commonly used in field trials as the desired level of statistical power. Many field trials study NTOs, with separate fields as replicates. Therefore, large numbers of replicates are needed over several seasons to test the hypotheses in the face of effect of confounding environmental variables. A power analysis indicated that replication of 20 experimental units (fields) per crop per year over 3 years (in total, 60 replicates) should yield adequate power (>80%) to detect differences of 1.5 fold or to detect 50% difference (Perry et al. 28). This minimum sample size will increase if the heterogeneity of the spatial distribution of the NTOs increases.

**Figure 4:**
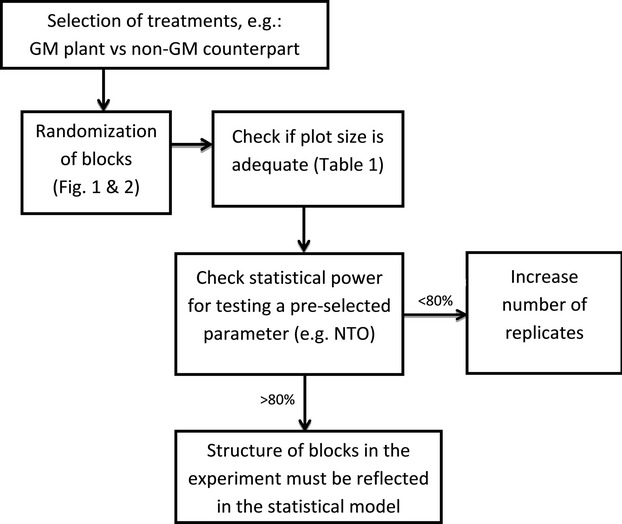
A scheme which helps to avoid the most common problems encountered with the setup of a field trial, based on the importance of statistical power.

For data that approximately follow a normal distribution, the power of standard tests (e.g., ANOVA) can be calculated routinely. Based on the assessment of statistical power for trial experiments, a simple scheme is proposed (Fig. 4) which can help to avoid the most common problems encountered with the setup of a field trial.

## Statistical Models for Data Analysis

Any data set obtained in an experiment has a particular distribution. It is the distribution of the data (i.e., the dependent variable or the response variable) that dictates what statistical tools are appropriate for use. Analysis of variance (ANOVA) can only be used in cases in which the data follow a normal distribution, possibly after applying a transformation, such as the logarithmic one (Fernandez 14; McCulloch 25; Zuur 40). ANOVA provides a statistical test of whether or not the means of several groups are equal, and therefore generalizes the *t*-test (test of the null hypothesis that the means of two normally distributed populations are equal) to more than two groups. The most common use of ANOVA is a linear relation of the response to the treatments and blocks. ANOVA should follow several assumptions: 1) independency of units (section 2.4); 2) the distributions of the residuals are normal; 3) equality (or “homogeneity”) of variances, and 4) the variance of data in groups should be similar. In case of skewed distributions of the data, restricted maximum likelihood has to be used (Bolker et al. 6).

There are three classes of models used in ANOVA, that is, fixed effects models (FEM), random effects models (REM), mixed models (MM).

### FEM, REM, MM

The statistical tools to be used are commonly aggregated under so-called models, like FEM, REM, MM, next to GLM and generalized linear mixed models (GLMM) (see below). A FEM is a statistical model that represents observed quantities (a numerical property that can exist as a magnitude or multitude) in terms of explanatory variables that are treated as if they were fixed (Zuur 40). The FEM applies to situations in which the experimenter applies one or more treatments to the subjects of the experiment (e.g., using two levels of herbicide vs. a control without herbicide) to see if the response variable values change (e.g., the level of beetles vs. the control). This is in contrast to REM in which explanatory variables might be treated as if they arise randomly. Such models (REM) assist in controlling for unobserved heterogeneity when this heterogeneity is constant over time and correlated with independent variables. However, REMs are rarely used in NTO studies, because these studies all have at least one fixed factor, that is, a factor of which the levels are experimentally determined, such as differences among treatments, the influence of GM plants on NTOs. An MM is a statistical model containing both fixed and random effects. MMs are particularly useful in settings where repeated measurements are made on the same statistical parameters or if other sources of random variation (e.g., site effects) need to be accommodated for. This means that, in most of the cases, the MM is an appropriate model for NTO studies, as NTOs are usually sampled over time in multiple plot replicates.

### GLM and GLMM

The GLM is a flexible generalization of ordinary linear regression and analysis of (co)variance. Sometimes the data can be transformed (e.g., a logarithmic transformation; Fernandez 14) to stabilize the variance. GLMs generalize linear regression by allowing a linear model to be related to the mean of the underlying distribution via a nonlinear link function (explained below) and by allowing the magnitude of the variance of each measurement to be a function of the mean. In addition to continuous data (data that are continuous in a selected range, e.g., pH or concentration of dissolved carbon), GLMs allow the modeling of discontinuous (count) data (e.g., numbers of beetles counted per area) and proportions as well as the cases when many zeros in a data set are present (which is often the case for NTO data, and is a complicating factor for the statistical analysis). While there are other approaches (e.g., Chi-square) to analyze count data, none of them can be efficient and flexible enough as GLM or GLMM, especially not if the testing needs to take into account the effect of additional covariates or random effects. Thus, Chi-square tests can handle only the most simple tests, for instance the comparison of two treatments without blocking and time continuity. This is rarely possible for properly designed field trials.

GLMs consist of three elements:

1. probability distribution such as the normal, exponential, binomial, Poisson etc.
2. The linear predictor, which is the quantity which incorporates information about independent variables (such as temperature, concentration of herbicides) that may have an influence into the model. It is related to the expected value of the data through the link function.
3. The link function (mathematical function that links response variables to predictors), which provides the relationship between the linear predictor and the mean of the distribution function. There are many commonly used link functions, and their choice can be somewhat arbitrary. The link function can linearize the expected response value as well as homogenize the (expected) variances.

Finally, the GLMM is a particular mixed model. It is an extension of the GLM, in which the linear predictor contains random effects (e.g., blocking) in addition to the usual fixed effects (e.g., the level of herbicide). These random effects are usually assumed to have a normal distribution. In the GLMM, it is numerically difficult to estimate parameters. Various so-called approximate estimation methods have been developed, but unfortunately none has good properties for all possible models and data sets (e.g., for ungrouped binary data). For this reason, numerical methods involving the Markov Chain Monte Carlo method (Berg 4) have increasingly been used, as increasing computing power and advances in methods have made them more practical. However, drawbacks exist here too, as the underlying distribution needs to be specified in advance.

### Overdispersion

Overdispersion is the condition by which the variability of data in a data set exceeds the variability expected under a particular probability distribution. Thus, data which are normally distributed are never overdispersed. However, overdispersion can occur in GLM in which the mean and variance are functionally dependent. Counts may exhibit more variability than is possible under the Binomial or Poisson probability models. McCullagh and Nelder (24) suggested that overdispersion may be the normal situation in many environmental studies (including effects of GM plants on NTOs) rather than being an exception. This might be due to the fact that experimenters resort to a small number of probability distributions to model their data. In most of the cases, this leads to the Binomial or Poisson distributions for counts. It is also important to choose a proper distribution model that permits higher dispersion if necessary, such as a Beta-binomial instead of Binomial model and a Negative Binomial instead of a Poisson model. Overdispersion can also occur due to an improper selection of independent variables (e.g., the concentration of herbicides vs. environmental parameters) and effects to model the data. Such cases must be solved by altering the set of effects and independent variables and not by selecting a different probability distribution for the data. In many cases (Schabenberger and Pierce 43), overdispersion might be addressed by addition of random effects and coefficients to the linear predictor of GLM. In general, low levels of overdispersion can be handled very well by inflating the variance function with a fixed factor (which itself is then called the dispersion parameter). This approach turns a GLM into a GLMM.

### Equivalence testing

In most field trials to study the impact of GM crops on selected variables like NTO's, a difference test is used (difference between effects of a GM and non-GM crop). From the statistical point of view, there are several major reasons why this common statistical procedure may require review.

The error of most concern in a difference test is of falsely inferring that no impacts (possibly indicating no hazards) exist where, in reality, there are. Because the traditional statistical null hypothesis is one of equality (no difference between GM and non-GM crop), this error is relatively difficult to estimate and/or set to a desired magnitude. This disadvantage is overcome by the equivalence test, sometimes referred to as a “proof of safety,” as here the null hypothesis is one of inequality, however, the error of most concern may not be assessed easily (Andow 2). The advantage of equivalence testing is therefore that the responsibility is placed back onto those who wish to demonstrate the safety of GM plants to do high-quality, well-replicated experiments with sufficient statistical power (Perry et al. 29).

Equivalence testing contrasts with other biological experimentation. In the past, the experimenter tested the null hypothesis of inequality between a GM organism (GMO) and its control, which needed to be actively disproved to reach the conclusion that the GMO is equivalent to the comparator. The null hypothesis of the equivalence test is “there is a difference between the GMO and its reference of a certain minimum size” against the alternative hypothesis “there is no or only a small difference between the GMO and its reference”. Therefore, in this testing procedure, a significant result (rejection of the null hypothesis) is required in order to conclude that the GMO and the reference are equivalent in their effects. For example, Marvier et al. (47) reported a meta-analysis of 42 field experiments that indicated that nontarget invertebrates were generally more abundant in *Bt-*cotton and *Bt-*maize fields than in nontransgenic fields managed conventionally with insecticides. However, in comparison with insecticide-free control fields, certain NTO taxa were less abundant in the *Bt* crop fields (Perry et al. 29). A successful test needs equivalence limits, which are difficult to select for NTO field studies (Van der Voet et al. 36). Equivalence limits could be estimated from field studies with concurrent reference varieties, which are typically the same studies in which also the GM and its non-GM counterpart are tested. Therefore, Van der Voet et al. (36) suggested to establish a two-step procedure, the first step being the setting of equivalence limits, the second their use for assessing equivalence.

Van der Voet et al. (36) suggested that statistical methodology should not be focused exclusively on either differences or equivalences, but should rather provide a better understanding within which the conclusions of both types of assessment are allowed (Van der Voet et al. 36). Both approaches are complementary: statistically significant differences may point at biological changes caused by the genetic modification, but may not be relevant from the viewpoint of ERA (Environmental Risk Assessment). Equivalence assessments may identify differences that could be larger than natural variation, but such cases may or may not indicate a true biological change caused by the genetic modification. On the other hand, Ward et al. (38) suggested that comparisons with traditional equivalence testing are not very helpful, because with such a test, the focus is on whether the difference between two treatments is less than a prespecified amount. A situation could arise in which two different submissions with very similar profiles (e.g., GM, comparator, and environment) could result in different conclusions because the respective sets of reference varieties led to different sets of equivalence limits (Ward et al. 38).

## Statistical Approaches and Real Data

In this section, various field experiments are discussed and the merits of the experimental designs or statistical analyses are highlighted (using Checklist for field trials, Table 2). This analysis may assist us in setting up new experiments and assessing the (biological) relevance of results of field trials with GM plants.

**Table 2:** Checklist for field trials that provides guidance in the use of statistical principles related to field testing of GM crops

Step	Explanation
1.	Statement of a hypothesis: in any field test, a hypothesis has to be formulated. As a hypothesis is a statement of the presumed relationship between variables, it must be properly stated. The hypothesis suggests a particular relationship between variables and it therefore narrows the problem to one that is specific and researchable. This makes the specification of independent and dependent variables relatively easy.
2.	Definition of variables: In order to observe whether the hypothesized relationship between variables exists, the latter must be clearly defined. Definition of the variables in a trial experiment allows everyone (both the experimenter and the regulator) to know what is being studied and facilitates interpretation of the results, thus, a description of the variables and the samples is required (e.g., what species, what larval stage, where were samples taken, when, and how).
3.	Specification of sample: The experimenter must clearly define which biological parameters (e.g., NTO) are studied and how: Were all possible or a specific set of NTOs studied? Were samples randomly selected? Was the sample only one organism or many? Were organisms made up in groups? These clarifications will help to determine the generalizations that were made, the data collection procedures that were selected, and the statistical analysis that was employed.
4.	Experimental design: The experimental design chosen should allow the experimenter to test the hypothesis. In the design, the experimenter should have provided answers to the following question and considerations: Were the treatments blocked? If not, then use completely randomized design. If yes and only one variable was studied, then use a randomized block design. If there was more than one variable, use a factorial randomized block design. When the experimental design is selected, the following questions have to be answered positively: 4.1. Were the treatments (blocks) properly randomized? (Fig. 1). 4.2. Were the treatments (blocks) properly replicated? (Fig. 2). 4.3. Was the experimental unit size appropriate for a certain NTO? (Table 1). Justification should be provided. 4.4. Was the sample size appropriate for a certain NTO? (2.3. Sample size). Sample size calculation (or justification) should be provided as well as timing, frequency, and duration. 4.5. Was true replication performed and pseudoreplication avoided? (Fig. 3). How were subsamples pooled?
5.	Statistical power: statistical power is the probability that the test applied will reject the null hypothesis when the null hypothesis is indeed false. It also provides the confidence that replication is neither too small to detect effects that are present, nor too great to avoid that unnecessary extra resources are used for trial experiments. Values of 70% (Prasifka et al. 42) and 80% (Perry et al. 28) are commonly used in field trials as the desired statistical power. The EFSA guidance document requires a prospective power analysis in order to test whether the design and the sample size will be able to test the hypotheses at hand.
6.	Statistical analysis: After the data have been collected, the experimenter must assess the relationships between independent and dependent variables. Most of these assessments are based on statistical analyses. *6.1. Type of null hypothesis: This hypothesis, denoted H_0_, should be capable of being proven false using a test of observed data. The null hypothesis typically corresponds to a general or default position. A set of data can only reject a null hypothesis or fail to reject it. Test of difference (H_0_: μ_1_ = μ_2_) or equivalence test (H_0_: μ_1_–μ_2_ > σ or H_0_: μ_1_–μ_2_ < −σ).* *6.2. What types of data were analyzed?* *6.2.1. If data are continuous (e.g., pH) then consider the normal or log-normal distribution (i.e., use a log transformation) and subsequently use ANOVA for balanced or REML for unbalanced (asymmetric) data. Check the residual plot.* *6.2.2. If data are counts (e.g., numbers of larvae), then GLM with Poisson distribution and log-link function are used. Either use a GLM for simple block design or a GLMM for designs such as split-plot design. In case of overdispersion, use a quasi-likelihood approach (i.e., variance proportional to the mean). An alternative way to model overdispersion is by using the negative binomial distribution.* *6.2.3. If data are proportions (e.g., the mortality of larvae), use GLM with binomial distribution and proper link function (e.g., logit). Either use a GLM for simple block design or a GLMM for designs such as split-plot design. In case of overdispersion of the data, use a quasi-likelihood approach. An alternative way to model overdispersion is by using the beta-binomial distribution. Overdispersion should not be used for 0/1 data as overdispersion is then not possible.* *6.3. Fixed and Random effects: these are the types of dependent variables in statistical analyses (Box 8). Check how the fixed and random effects were selected.* *6.4. Overdispersion: is the condition by which the variability of the data exceeds that expected under a certain probability distribution (data which are normally distributed are never overdispersed).* *- Check for overdispersion (the occurrence of more variance in the data than predicted by a statistical model), especially for data that follow a Poisson (counts) or binomial distribution (proportions) (Box 10).* *- In some cases, distribution models might have to be changed to Beta-binomial instead of Binomial and Negative Binomial instead of Poisson to deal with overdispersion. Overdispersion might also be addressed by the addition of random effects and coefficients to the linear predictor of GLM. This approach turns GLM to GLMM.*

Most of the mistakes discussed in Section 5 can be avoided if the rules below are considered.

**Figure 5:**
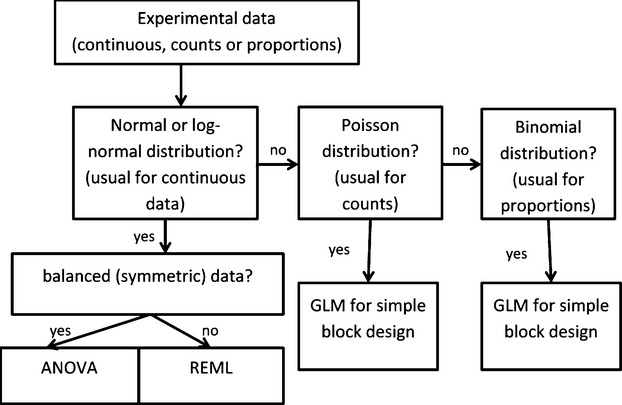
Initial structure of statistical considerations that helps to select the appropriate test

A first example is offered by a study by Hoss et al. (17). They assessed the possible influence of GM maize (expressing the insecticidal Cry3Bb1 protein), as compared to non-GM maize, on the abundance of free-living soil nematodes. While the experimental design (randomized complete block design) was fair for such a comparison (steps 1, 2, 3, and 4 in Table 2), the statistical approach was rudimentary for its purpose. An ANOVA with maize cultivar as the fixed factor and block as a random factor (6.3. in Table 2) was carried out to test for differences in the measured parameters (nematode counts) between the two cultivars. As the measurements encompassed discontinuous (count) data, the use of a GLM on the basis of a Poisson distribution would have been appropriate (Premise 6.2.2. in Table 2 is violated). Count/Poisson-distributed data have the property that the variance increases with the mean, which violates the ANOVA assumption of homogeneous variances. Thus, applying ANOVA to such data can lead to inaccurate p-values. Moreover, an analysis of the statistical power of the test was not performed (5. in Table 2).On the basis of this, we propose the initial structure of statistical considerations that guide us to the appropriate test shown in Fig. 5.

Another example of fairly improper use of statistics can be found in Oliveira-Filho et al. (27). These authors studied the influence of pest control agents on nontarget invertebrates in soil. For comparison of the treatments, they transformed the count data of invertebrates to percentages of a maximum (to distinguish relative changes), after which differences in the percentages between the treatments were evaluated by one-way ANOVA. The authors did not check in their data whether the variance increased with the mean. As in the above, a GLM (for proportions) instead of ANOVA (for continuous data) should have been used (Premise 6.2.3. in Table 2 is violated). A similar problem can be found in Post and Parry (30). These authors studied the effects of transgenic blight-resistant American chestnut, as compared to a non-GM variant, on insect herbivores in a completely randomized block design (4. in Table 2). Although it was appropriate to use one-way ANOVA for comparisons of the growth rates (continuous data) of insect herbivores (6.2.1.in Table 2), the use of this test to compare the counts of the insect herbivores was not (6.2.2.in Table 2 is violated). Again, the use of a GLM on the basis of the Poisson distribution would have been indicated. As we can see from these examples, usage of the above decision tree or Checklist for field trials (Table 2) assists us in selecting the appropriate statistical analyses.

Furthermore, a farm-scale study (Spain) was initiated in 2000 to assess the potential impact of *Bt*-maize on the abundance and diversity of predatory arthropods (de la Poza et al. 31). The experimental setup was a randomized block design (4. in Table 2)involving three treatments, each with four (Lleida) or three (Madrid) replicates. The treatments were: (1) *Bt* transgenic maize, (2) the isogenic hybrid without insecticide treatment, and (3) the isogenic hybrid with imidacloprid insecticide seed treatment. In the combined ANOVA, a split-plot model was used, in which year and location were considered as the main plots. Subplots were the treatment (3 treatments) and sub-subplots were the sampling dates. All factors, except blocks, were considered to be fixed and crossed with each other, except, again, for blocks that were nested within locations and years (6.3. in Table 2). A priori comparisons of the means among treatments within a given environment (year per location combinations) were performed with the adjusted least square means, using standard t-tests. To normalize the original data, these were transformed by square root transformation prior to analysis (6. in Table 2). This study thus used the appropriate statistical tools.

In the case of Druart et al. (10), who studied the influence of pesticide drift and transfer on nontarget snails in soil, the experimental design (steps 1, 2, 3, and 4 in Table 2) and statistical analysis were also quite adequate. Differences in snail mass or shell diameter were assessed by a linear mixed effects model with zone as the fixed explanatory variable and microcosm as the random variable (6.3. in Table 2). The mortality for each treatment between each zone and mortality between treatments in all zones (pooled) were assessed by a binomial GLM (see the tree above) (6.2.3. in Table 2), resulting in an appropriate statistical analysis.

Proper statistical analysis can also be applied even if a relatively complex experimental design is used. Thus, Stoleson et al. (35) studied the responses of bird communities to an operational herbicide treatment over time. They used a randomized block design (4. in Table 2), in which half of each 8-ha block received herbicide and the other half acted as control. As for the statistical analyses, they used GLMMs to model the effects of year, site, herbicide treatment and cutting sequence on vegetation and avian target variables (6. in Table 2). In all models, they considered site as a random effect and year, herbicide treatment, and cutting sequence as fixed effects (6.3. in Table 2). Shannon indices were modeled using a Beta distribution, while other diversity indices were modeled with a normal distribution. Vegetation covers were modeled using a log-normal distribution, whereas bird abundances were modeled using a Poisson distribution. All models used the restricted maximum likelihood method. While standard maximum likelihood (used for classical ANOVA to fit parameters) estimates the standard deviations of the random effects assuming that the fixed-effect estimates are precisely correct, restricted maximum likelihood averages some of the uncertainty in the fixed-effect parameters to adjust the denominator degrees of freedom. The analyses of species diversity indices are confounded by effects over different spatial scales (Crist et al. 41). However, diversity indices are not recommended for general GM plant impact assessment and univariate statistics (e.g., the presence or density of a NTO) or multivariate approaches may be more appropriate (Perry et al. 29).

**Figure 6:**
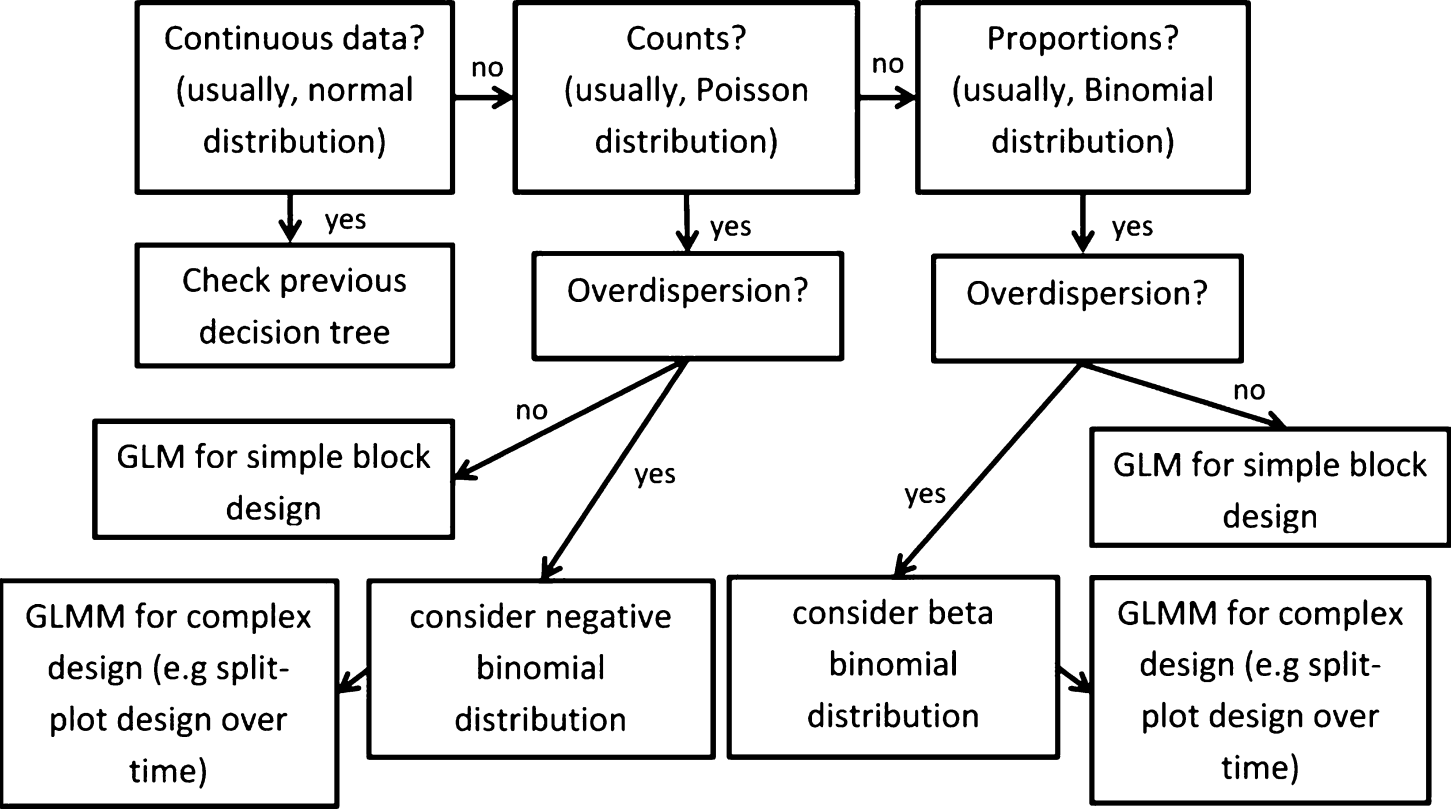
The improved structure of statistical considerations for the most common part (count and proportion data for NTO) of the decision tree, based on the discussed weaknesses and strengths of the methods.

On the basis of the discussed weaknesses and strengths of the methods used in the above described examples, we modified the most common part (count and proportion data for NTO) of the decision tree (Fig. 6).

From all examples one may conclude that, when applying significance tests for abundance data, we often face the problem of uncertainty concerning the true effect (or the width of confidence intervals, in case of equivalence tests). This may become large for low abundances (power analysis can provide insight into the size of the problem), small numbers of replications and large residual variation. Counting of individuals scattered randomly in the observational windows might yield data following the Poisson distribution. However, large residual variation in abundance data often occurs due to the clustering of individuals, termed extra-Poisson variation or overdispersion (McCullagh and Nelder 24). Therefore, the probability to find a significant difference in the case that the GM plant has no effect on the abundance of a species (Type I error) may be high if rare species or species with high variability in local or temporal abundance or activity are investigated with a low number of replications. On the other hand, several types of NTOs (e.g., collembola) might be characterized as types for which a relatively low number of replications (due to their high density) is sufficient. Thus, planning trials with a sufficient number of replications, based on available prior information concerning the mean abundance and variability of the observations is an important issue. For this purpose, we suggest to analyze available datasets of a certain NTO (possibly obtained from other field trials) concerning its mean abundance and variability. In complex cases, it is important to simulate abundance data for different choices of mean abundance, variability, and experimental design.

## Discussion

This review summarizes the most important statistical considerations with respect to the field testing of GM (mainly insect-resistant) crop plants in relation to their potential effects on NTOs. It is important to carefully consider the following issues:

1. The objective of the study and the thus required experimental setup (see 3),
2. Experimental unit (field) size and its implication for NTO impact testing,
3. Experimental setup, including design, randomization, and replication,
4. Statistical power testing,
5. Type of the frequency distribution of the dependent variable,
6. Potential overdispersion of the data and implications for statistics,
7. Difference versus equivalence testing.

In the light of these points, it is essential for experimenters to plan field experiments in the most optimized way concerning the expected types of effects, data, and variability. Without a clear prior understanding of the experimental hypothesis and how the results will be analyzed and interpreted, an incorrect statistical analysis may be applied, which will lead to incorrect conclusions. Next to considering the importance of proper randomization and replication, avoiding pseudoreplication, an experimenter has to pay extra care to the specific rules that apply to GM plant field testing, such as the size of the plots and consequently the size of the field required, to test for impacts on NTOs. A common aspect of current GM plant field trials is the fact that fields are too small to adequately assess the impact on NTOs in a robust manner. Moreover, the statistical power of the design has to be checked routinely in order to be sure that the experimental trials are appropriate (e.g., the number of replicates and the sample number per replicate). Considering existing field experiments, there are many examples (Section 5) in which improper statistical analyses have been applied. The most common flaw is that discontinuous data (counts) are analyzed by ANOVA. This in spite of the fact that only generalized linear models (GLM) are found to be efficient enough for analysis of these kinds of data. There is a Chi-square test that has been in use to analyze count data, but it is not efficient and flexible enough as compared to GLM or GLMM. Chi-square tests can handle only the most simple tests, such as the comparison of two treatments without blocking and time, which is rarely possible for properly designed GM plant field trials. Recently, equivalence and difference testing have been proposed as appropriate approaches to deal with NTO impact data from GM plant field trials (Perry et al. 28). There is no *a priori* scientific justification for either of the two approaches, and hence it can be argued that usually difference testing is as appropriate as equivalence testing, if both the experimental design and statistical analyses are justified. Moreover, it is difficult to analyze discontinuous data by equivalence testing for nonprofessional statisticians.

The guidance document by EFSA (12) attempted to harmonize approaches but it does not provide clear and structured suggestions for statistics of field trials. The use of the proposed decision trees and the “Checklist for field trials” (Table 2) offers a reasonable approach and solution to avoid improper statistical analyses. In particular, we would like to stress that they highlight ways to overcome or avoid the many common severe problems in the final interpretation of the results of treatment comparisons in field trials. Only professional statisticians are normally able to provide the certainty that all steps of a chosen statistical approach are taken in a proper way, as minor details or deviations can lead to incorrect final conclusions. However, statisticians are still in debate about the most proper statistical approaches for assessments of GM and reference plant varieties, in particular when it comes to NTO impact assessments (Ward et al. 38).
